# Nanoparticle Drones to Target Lung Cancer with Radiosensitizers and Cannabinoids

**DOI:** 10.3389/fonc.2017.00208

**Published:** 2017-09-19

**Authors:** Wilfred Ngwa, Rajiv Kumar, Michele Moreau, Raymond Dabney, Allen Herman

**Affiliations:** ^1^Department of Radiation Oncology, Dana-Farber Cancer Institute, Brigham and Women’s Hospital, Harvard Medical School, Boston, MA, United States; ^2^University of Massachusetts Lowell, Lowell, MA, United States; ^3^Northeastern University, Boston, MA, United States; ^4^Cannabis Science Inc., Irvine, CA, United States

**Keywords:** smart nanoparticles (drones), radiotherapy, radiosensitizers, cannabinoids, inhalation, intravenous delivery, lung cancer, therapeutic efficacy

## Abstract

Nanotechnology has opened up a new, previously unimaginable world in cancer diagnosis and therapy, leading to the emergence of cancer nanomedicine and nanoparticle-aided radiotherapy. Smart nanomaterials (nanoparticle drones) can now be constructed with capability to precisely target cancer cells and be remotely activated with radiation to emit micrometer-range missile-like electrons to destroy the tumor cells. These nanoparticle drones can also be programmed to deliver therapeutic payloads to tumor sites to achieve optimal therapeutic efficacy. In this article, we examine the state-of-the-art and potential of nanoparticle drones in targeting lung cancer. Inhalation (INH) (air) versus traditional intravenous (“sea”) routes of navigating physiological barriers using such drones is assessed. Results and analysis suggest that INH route may offer more promise for targeting tumor cells with radiosensitizers and cannabinoids from the perspective of maximizing damage to lung tumors cells while minimizing any collateral damage or side effects.

## Introduction

Nanomedicine, the application of nanotechnology to medicine, has opened a new, previously unimaginable world in cancer diagnosis and therapy. Today new multifunctional nanoplatforms or smart nanomaterials (nanoparticle drones) can be constructed and endowed with image contrast enhancement capabilities for techniques such as computed tomography (CT) and magnetic resonance imaging (MRI) (1, 2) and can contain therapeutic payloads programmed for targeted delivery to disease sites (3). The vision of combining diagnostics and therapeutics, now being referred to as theranostics, was considered futuristic only a few years ago but is now clearly achievable—the future is almost now!

Recognizing the potential impact of nanomedicine, the National Cancer Institute created the Alliance for Cancer Nanotechnology to leverage the potential of nanotechnology toward transforming the way cancer is diagnosed, treated, or prevented. Projects funded by this Alliance have led to significant research breakthroughs and have even entered successful clinical trials (4). Today, cancer nanomedicine now includes burgeoning research and development in nanoparticle-aided radiotherapy (NRT). A recent article (5) provides a robust review of NRT developments for over a decade in NRT with gold nanoparticles (GNPs), highlighting emerging approaches, challenges, and opportunities for further research toward clinical translation. Beyond GNP, other research has highlighted the use of alternative nanoparticle platforms like gadolinium nanoparticles (6, 7), hafnium nanoparticles (8), platinum-based chemotherapy drug platforms, and others with theranostic capability (9, 10).

In general, the key goal for NRT and cancer drug development efforts is the same, which is to optimize therapeutic efficacy/ratio. To this end, recent advances in the design of smart nanomaterials proffer tremendous potential toward realizing this goal. Such smart materials (11) are specifically designed to be sensitive to a specific stimulus, such as temperature, magnetic field, ultrasound intensity, light or radiation, and pH, and to then respond in active ways including radiosensitization or changing their structure for drug delivery, or other functions that have the potential to cogently enhance treatment outcomes.

Gold nanoparticles provide an excellent template for building such nanoparticle drones. They are biocompatible radiosensitizers (5), proffering relatively no toxicity. They can readily interact with photons by the photoelectric effect, to emit missile-like photoelectrons or Auger electrons in the micrometer range, to substantially boost RT damage to cancer cells. In the photoelectric effect, photons interact with the nanomaterials, with the probability of photoelectric interaction inversely proportional to the cube of the photon energy (5). Once the photoelectron is emitted, this creates a vacancy that may be filled by an electron from a higher energy level. The resulting release of energy could then also knockout Auger electrons. The Auger electrons are shorter range and with high linear energy transfer, so can lead to highly localized damage. Such highly localized damage to tumor cells can allow minimization of the primary radiotherapy dose and hence normal tissue toxicity. Nanoplatforms such as GNPs are also particularly attractive for building nanoparticle drones because they can provide CT and photoacoustic imaging contrast and are suitable for drug loading and attaching targeting moieties. Depending on surface functionalization, type of drug, and desired application, GNPs can be easily loaded with drugs or other molecules through either non-covalent interactions or covalent conjugation. Loading of drugs onto GNPs may improve their stability and biodistribution in biological media since the drugs are protected in the carrier. In short, multifunctional nanoparticle drones based on GNPs hold great promise in cancer nanomedicine.

## Challenges and Opportunities in Cancer Nanomedicine and NRT

Despite advances in cancer nanomedicine, a major challenge is to ensure that a sufficiently potent concentration of the nanoparticles or drug payload reaches the disease site with minimal systemic or off-target toxicities. In general, nanomedicine holds advantage over conventional medicine because the enhanced permeability and retention effect allows preferential delivery of payloads to tumor sites, and the ability to sustainably release such payloads overtime once they reach the tumors. The challenge to deliver sufficiently potent payloads is particularly relevant for external beam NRT (5), with a lower fraction of kilovolt energy photons, which can interact with nanoparticles to activate the emission of micrometer-range electrons. The challenge to deliver potent concentrations of drug payloads with minimal side effects is particularly relevant to delivery of drugs like cannabinoids whose clinical translation has been hampered by the psychotic side effects (12). New treatment strategies are, therefore, needed to overcome these challenges and improve therapeutic efficacy, allowing enhanced tumor cell killing while sparing normal tissue.

## Perspectives

One strategy to consider is the route by which nanoparticle drones with radiosensitizers or drugs can navigate physiological barriers to reach the tumor with minimal systemic toxicities. This will depend on the tumor site. One tumor site that could significantly benefit from new strategies is lung cancer, the leading cause of cancer deaths. Two key routes for targeting nanoparticle drones to lung tumors are inhalation (INH or “air”) route and intravenous (IV or “sea”) route, i.e., through the blood stream.

Previous studies show that administering nanoparticles *via* IV route may result in up to 5% nanoparticles reaching the lung, compared to 3.5–14.6 times higher nanoparticle concentrations when administered *via* INH (13, 14). Taking this into account, we have previously investigated the potential for enhancing external beam radiotherapy for lung cancer using high-Z nanoparticles (made of gold or platinum-based chemotherapy drugs) administered *via* INH (15). The results of this work indicate that administering nanoparticles *via* the INH route could enable clinically significant damage enhancement to lung tumors compared to using IV routes of administration during external beam radiotherapy for lung cancer. Building on this work, we conducted additional experiments using nanoparticle drones based on GNP using transgenic mouse models.

The design of such nanoparticle drones described in our previous work (16) particularly takes size and nanoparticle functionalization into account. The size is optimized to ensure increased circulation and tumor uptake. Meanwhile, PEGylation confers stealth to the nanoparticle drones, thereby, also enabling longer circulation time for nanoparticles administered intravenously. Such longer circulation allows for higher amounts of nanoparticles to concentrate in the tumor. The hetero-bifunctional-polyethylene glycol with amine, carboxyl, and methoxy ligands, also allows for conjugating various moieties such as imaging or targeting ligands, as well as radiosensitizers or drugs. The imaging capability of these nanoplatforms has already been demonstrated *in vitro* and *in vivo* (16). A number of recent efforts have also developed nanoparticle drone platforms, including biogenic GNPs (17, 18) and drug-loaded gold plasmonic nanoparticles (19, 20) for different applications. For lung tumors, the nanoparticle drones are functionalized with RGD designed to target the integrin receptors on the lung tumor.

**Figure 1:**
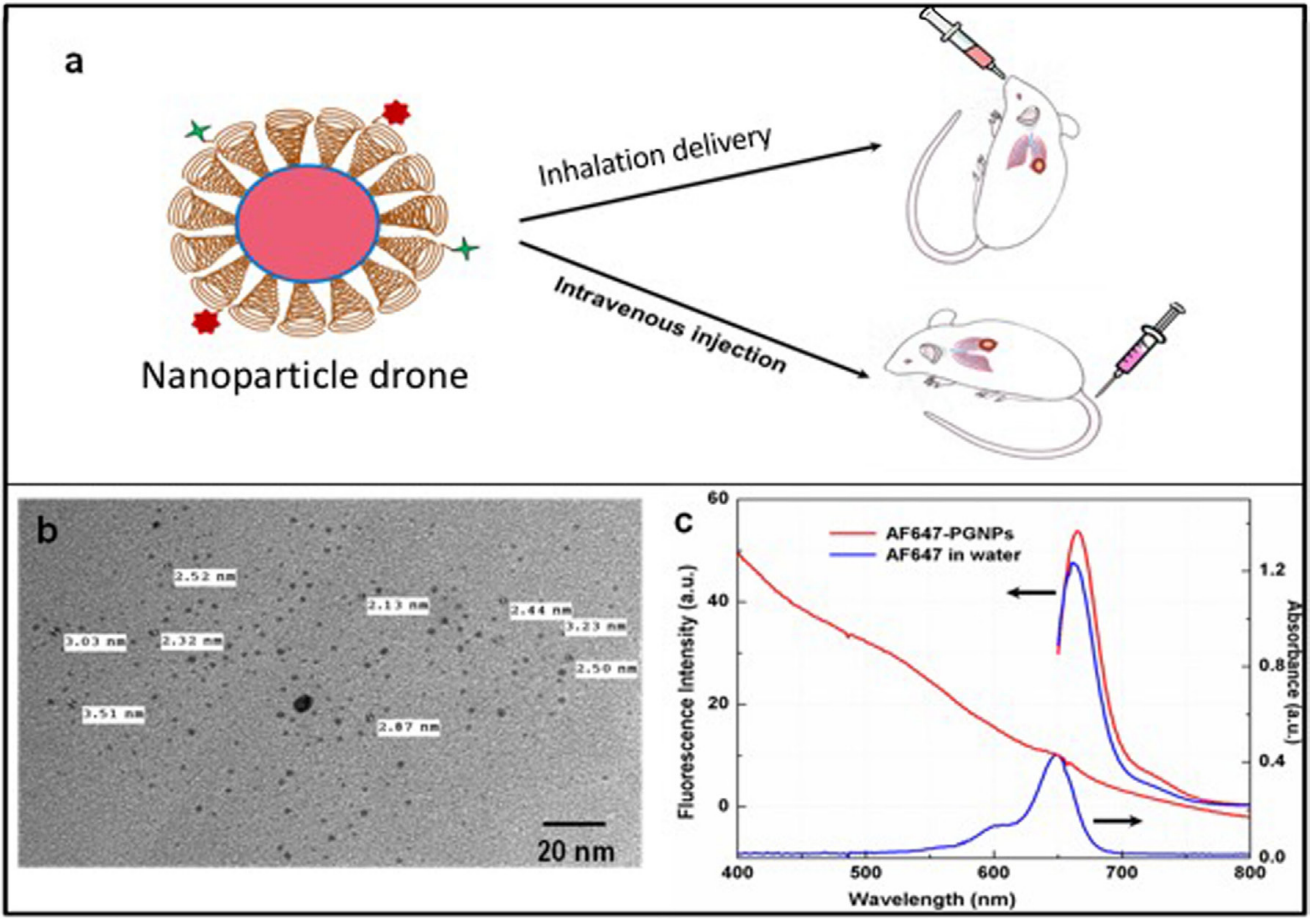
**(A)** Cartoon showing both intravenous and inhalation (INH) delivery of nanoparticle drones; **(B)** TEM image of lung tumor targeted with drones; **(C)** absorption spectra of drone technology uniquely customized for INH delivery to lung tumors.

In experiments, transgenic mouse models bearing single-nodule lung adenocarcinoma were employed, with features that closely resemble human lung tumors, as described in recent work (21). Nanoparticle drones were administered to Cohort A mice (*n* = 5) *via* INH (instillation), while the same concentration of nanoparticle drones was administered to Cohort B (*n* = 5) mice *via* IV (Figure 1). The biodistribution of drones was measured *via* fluorescence imaging and *ex vivo* electron microcopy methods. Mice were dissected, and entire lungs were imaged 24 h post administration. The images were acquired with the same acquisition time of 88 ms for all the experiments. With spectral imaging software, small but meaningful spectral differences could be rapidly detected and analyzed. Spectral unmixing algorithms were employed to generate unmixed images of “pure” autofluorescence and “pure” fluorescence signal. A quantitative estimation of the fluorescence intensity was done using the Maestro software and Image J.

Meanwhile, transmission electron microscopy was carried out using a JEOL model JEM-1000 microscope at an acceleration voltage of 80 kV. The samples were prepared by drop casting method on the formvar coated copper grids. 1 mm^3^ of lung tissues were fixed in 2.5% formaldehyde and 2.5% glutaraldehyde solution for few hours. The post fixation was carried out using 1% osmium tetraoxide for 1 h followed by dehydration in varying alcohol concentrations and overnight infiltration using Squetol resin. The polymerized resin with tissue was sectioned using ultramicrotome. The sections were placed on copper grids, and silver enhancement was done following the manufacturer’s recommendations. The grids were dried and imaged.

After each study, animals were euthanized by CO_2_ INH followed by cervical dislocation. Death was assured by harvesting tumor-bearing and other vital organs, including the cecum, liver, and lungs. All studies followed Dana-Farber Cancer Institute IACUC approved protocol.

The schematic (Figure 1A) illustrates the nanoparticle drone and the two different routes of administration in the transgenic lung tumor mouse model. The size of the nanoparticles as estimated by TEM (Figure 1B) showed fairly monodispersed nanoparticles of around 2–3 nm in diameter with spherical morphology. The overall hydrodynamic diameter of the drones was approximately 12 nm as estimated by the dynamic light scattering measurements (data not shown). Meanwhile, Figure 1C shows the photophysical characteristics of the nanoparticle drones. As the figure shows, drones conjugated with AF647 showed a characteristic fluorescence peak at λ_max_ 650 nm with a slight bathochromic shift of 5 nm. This can be attributed to slight aggregation or steric hindrance of the dye molecules after conjugation to PEG. The absorption studies showed a slight hump in the nanoparticle drone spectra at around 647 nm. However, a distinct absorption peak is masked due to higher degree of scattering by the GNP drones.

**Figure 2:**
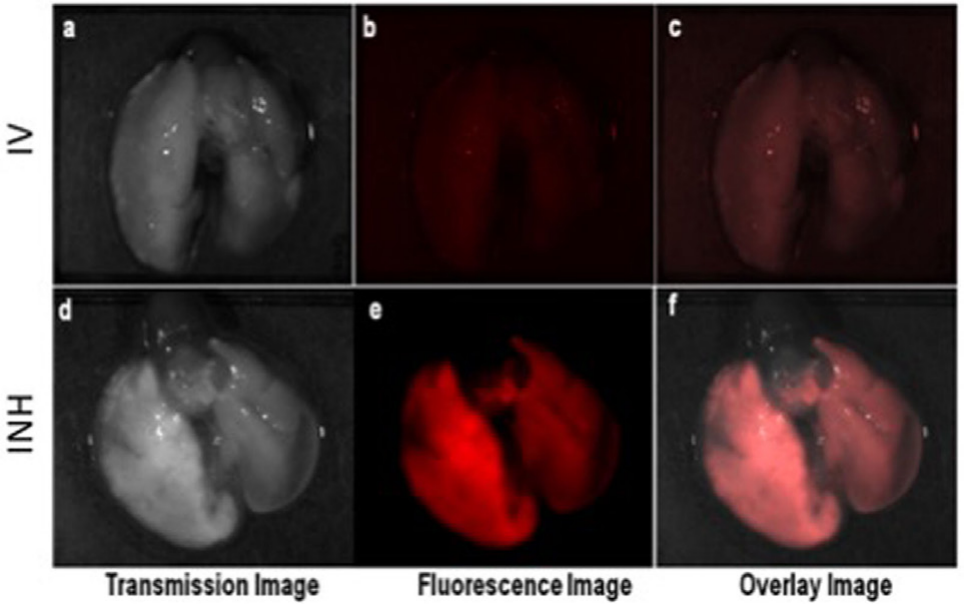
*Ex vivo* optical studies with the lung dissected from the mice administered with nanoparticle drones. Fluorescent imaging illustrating the distribution of fluorescent drones within the mouse lung administered *via* intravenous (IV) in panels **(A–C)** and inhalation (INH) in panels **(D–F)**.

**Figure 3:**
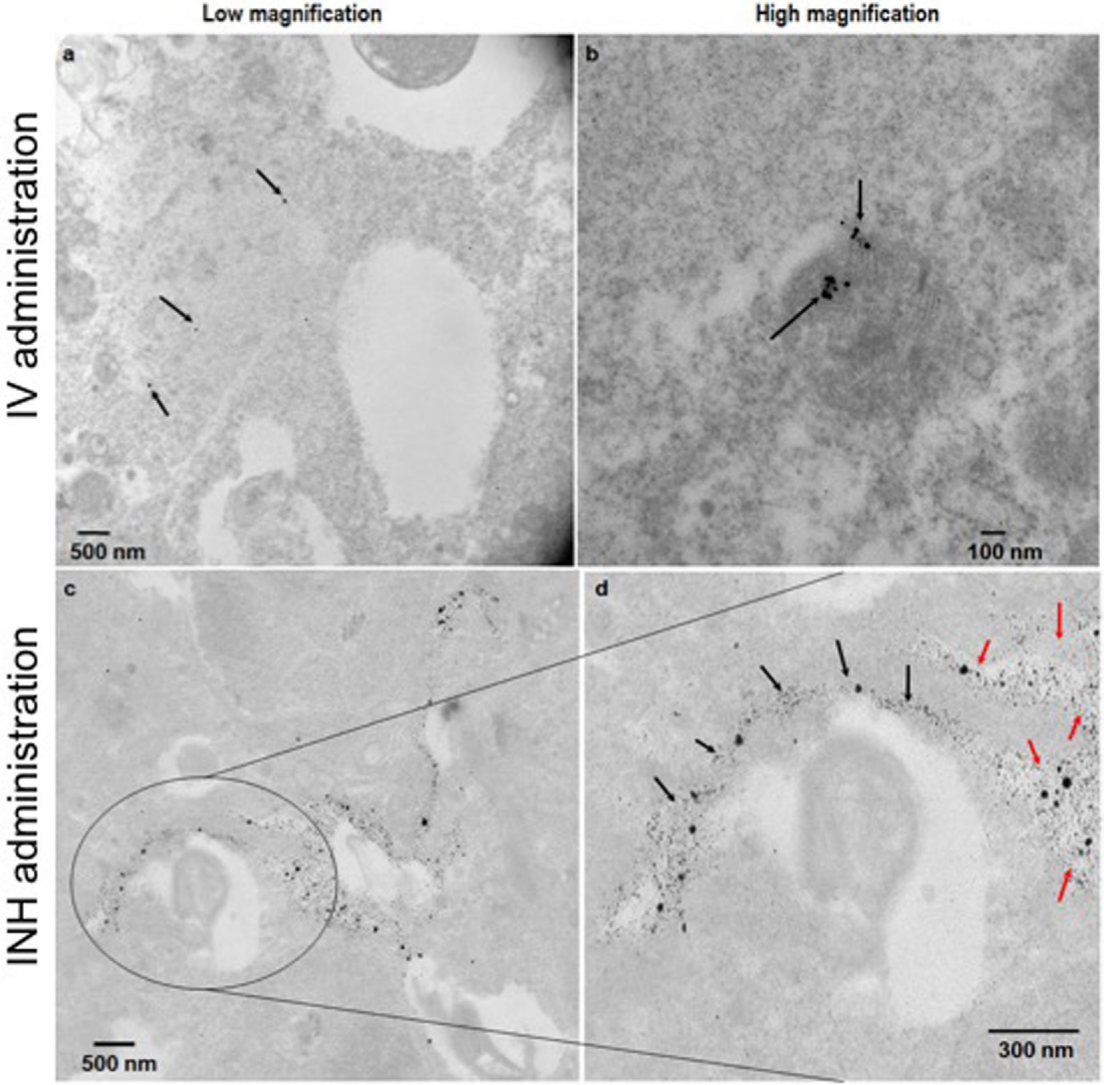
TEM images of the lung tumor sections with nanoparticles drones administered intravenously **(A,B)** or *via* inhalation (INH) route **(C,D)**. Panel **(D)** is a magnified area of the image in panel **(C)** to show the nanoparticles drones that have migrated from the air spaces (red arrows) to the tumor tissue (black arrows).

Figure 2 shows the comparative imaging studies with the nanoparticle drones administered *via* INH or IV routes at similar concentrations. The transgenic lung tumor model, which we used for this study, develops tumor only in the left lung (21). Figures 2A,B show white light optical images of sample lung tumoral masses with drones reaching their target after administration *via* INH and IV, respectively. The corresponding fluorescent images are shown in Figures 2C,D, respectively. From these images, significantly higher fluorescence due to the drones was consistently observed for the left lung in case of INH administration compared to IV. This is further corroborated by the TEM images in Figures 3A,B for IV and Figures 3C,D for INH. Consistent with the findings from fluorescence imaging, the TEM images show nanoparticle drones abundantly distributed throughout the target tumor tissue in mice with INH route of delivery compared to sparse distribution in sample tissue sections of mice with IV route.

For more perspective, the pixel intensity for randomly selected regions of interests was quantified and compared. From the quantification, the drones reaching their target tumors *via* the INH (air) route were averagely about 4.8 ± 0.4 times higher, compared to IV (“sea”) route. This finding for nanoparticle drones is consistent with the previous studies showing that INH route provides 3.5–14.6 times higher nanoparticle concentrations compared to IV (13, 14). In our study, the nanoparticle drones can also be visualized penetrating into the tumor tissue. This can be attributed to the small size of drones (<100 nm), which facilitates enhanced diffusion and adsorption and also targeting by RGD peptide. Larger nanoparticles are known to deposit in the extrathoracic and bronchial region airways during inspiration. Altogether, the results support the perspective that nanoparticle drones administration *via* INH could lead to significantly higher targeting to lung tumors compared to IV routes. Our previous work (15) has showed that this could lead to a clinical significant radiotherapy boosting to lung tumor cells compared to IV routes.

## Potential for Cannabinoids

As discussed earlier, nanoparticle drones are particularly attractive because they can also be loaded with drugs payload like cannabinoids. Cannabinoids, which are the bioactive components of *Cannabis sativa* and their derivatives, may exert palliative effects in cancer patients by preventing nausea, vomiting, and pain and by stimulating appetite (12). Furthermore, studies indicate that cannabinoids can inhibit cancer cell growth in *in vitro* and *in vivo*. A Nature Reviews Cancer article (12) and other recently published work (22–24) highlight the potential of cannabinoids for treating cancer, working in synergy with radiotherapy and serving as radiosensitzers to enhance damage to lung tumor cells in particular. Consistent with this, our own experiments have confirmed the potential of cannabinoids in treating lung cancer, with results confirming that cannabinoids can enhance damage to cancer cells. In one study, the damage to lung tumor cells was found to be similar to that equivalent to 4 Gy of radiotherapy dose. Results indicate that the interaction can be synergistic with radiation. Such synergy with radiotherapy was seen when cannabinoid concentrations of 2 µg/ml were used. Previous studies have also shown synergistic interaction of cannabinoids with radiotherapy *in vivo* (23).

Despite this growing evidence with promising *in vitro* and *in vivo* studies, clinical translation of cannabinoids is severely limited by unwanted psychoactive side effects (12). Therefore, cannabinoid-based therapies or strategies that could circumvent these unwanted psychotic side effects are greatly needed. For lung cancer, the use of nanoparticle drones may provide an excellent strategy for highly targeted delivery of cannabinoid payloads to tumor cells to boost damage to the tumor cells and inhibit growth while minimizing the side effects that have hampered clinical translation.

In using cannabinoid-loaded nanoparticle drones, the cannabinoids can be readily conjugated to the amine groups present on the PEG on the surface of the GNPs (22, 25, 26). Recent work has also highlighted the use of cannabinoid-loaded polymer-based microparticles to inhibit tumor growth in a murine xenograft model of glioblastoma multiforme (27). Such polymers can also be used for nanoparticle drones encapsulating cannabinoids for sustained delivery to tumor cells. In the work with microparticles, they were administered locally (28). For lung tumors, INH route provides advantage in delivering such cannabinoid payloads.

Alternative approaches to get cannabinoids to tumors include intratumoral administration (27). Such an approach will ensure virtually all the payload reaches the tumor. However, for lung tumors, this is an invasive procedure, which may generate significant morbidities. Another approach could be *via* smoking. Unfortunately, smoking is already associated with lung cancer and other side effects (29), and smoking does not allow for any additional targeting of the cannabinoids to lung tumors as with targeting moieties like RGD used on nanoparticle drones. However, comprehensive studies are needed to establish the envisioned advantage of using nanoparticle drones administered *via* INH.

Today, over 26 USA states, and an increasing number of countries have now legalized medical cannabis for treating different malignancies. The National Institutes of Health and World Health Organization encourage more research on medical cannabis for treating cancer, the side effects of radiotherapy or chemotherapy, and treatment of other diseases. With ever increasing interest in medical cannabis applications in the USA and around the world, and promising research results there is great need for more concerted cross-disciplinary research collaborations, including academic institution–industry collaborations, which could accelerate medical cannabis research from bench to bedside.

## Conclusion

Overall, the use of nanoparticle drones administered *via* INH to enhance NRT, as highlighted in this article, may provide a good strategy for maximizing therapeutic efficacy in external beam NRT for lung cancer. Also there is growing evidence that cannabinoids can serve as radiosensitizers, enhance damage to tumor cells, slow tumor growth, and work synergistically with radiotherapy in cancer treatment. There is growing consensus of the need for more research in this direction, including research to address unwanted psychoactive effects, which currently limit clinical translation. The use of nanoparticle drones *via* INH provides a promising strategy for consideration in overcoming this limitation by delivering sufficiently potent cannabinoids to lung tumors while minimizing toxicities or side effects. Ongoing studies are investigating the optimization of nanoparticle drones for highly efficacious targeting of lung tumors with radiosensitizers or cannabinoids while minimizing collateral damage or side effects. This perspective article should motivate more interdisciplinary collaborations and concerted research efforts in this direction toward advancing the promise of cancer nanomedicine to benefit patients with lung cancer.

## Ethics Statement

Results on animal work highlighted in this article were approved by the Dana-Farber Cancer Institute IACUC.

## Author Contributions

RK contributed in design and testing of nanoparticle drones highlighted in this article. MM contributed in generating results with cannabinoids highlighted in this article. AH reviewed the manuscript and provided intellectual contributions. RD reviewed the manuscript and discussed the perspective on using nanoparticle drones loaded with cannabinoids. WN wrote the manuscript highlighting the perspective of using inhalation route for targeting nanoparticle drones to lung tumors to boost therapeutic efficacy and ratio.

## Conflict of Interest Statement

Coauthors RD and AH work for the for-profit company Cannabis Science Inc. All other authors declare that the research was conducted in the absence of any commercial or financial relationships that could be construed as a potential conflict of interest.
